# The Effect of a WeChat-Based Tertiary A-Level Hospital Intervention on Medication Adherence and Risk Factor Control in Patients With Stable Coronary Artery Disease: Multicenter Prospective Study

**DOI:** 10.2196/32548

**Published:** 2021-10-27

**Authors:** Boqun Shi, Xi Liu, Qiuting Dong, Yuxiu Yang, Zhongxing Cai, Haoyu Wang, Dong Yin, Hongjian Wang, Kefei Dou, Weihua Song

**Affiliations:** 1 Cardiometabolic Medicine Center, Fuwai Hospital National Center for Cardiovascular Diseases Chinese Academy of Medical Sciences and Peking Union Medical College Beijing China

**Keywords:** WeChat, telemedicine, coronary artery disease, medication adherence, mobile phone

## Abstract

**Background:**

In China, ischemic heart disease is the main cause of mortality. Having cardiac rehabilitation and a secondary prevention program in place is a class IA recommendation for individuals with coronary artery disease. WeChat-based interventions seem to be feasible and efficient for the follow-up and management of chronic diseases.

**Objective:**

This study aims to evaluate the effectiveness of a tertiary A-level hospital, WeChat-based telemedicine intervention in comparison with conventional community hospital follow-up on medication adherence and risk factor control in individuals with stable coronary artery disease.

**Methods:**

In this multicenter prospective study, 1424 patients with stable coronary artery disease in Beijing, China, were consecutively enrolled between September 2018 and September 2019 from the Fuwai Hospital and 4 community hospitals. At 1-, 3-, 6-, and 12-month follow-up, participants received healthy lifestyle recommendations and medication advice. Subsequently, the control group attended an offline outpatient clinic at 4 separate community hospitals. The intervention group had follow-up visits through WeChat-based telemedicine management. The main end point was medication adherence, which was defined as participant compliance in taking all 4 cardioprotective medications that would improve the patient’s outcome (therapies included antiplatelet therapy, β-blockers, statins, and angiotensin-converting-enzyme inhibitors or angiotensin-receptor blockers). Multivariable generalized estimating equations were used to compare the primary and secondary outcomes between the 2 groups and to calculate the relative risk (RR) at 12 months. Propensity score matching and inverse probability of treatment weighting were performed as sensitivity analyses, and propensity scores were calculated using a multivariable logistic regression model.

**Results:**

At 1 year, 88% (565/642) of patients in the intervention group and 91.8% (518/564) of patients in the control group had successful follow-up data. We matched 257 pairs of patients between the intervention and control groups. There was no obvious advantage in medication adherence with the 4 cardioprotective drugs in the intervention group (172/565, 30.4%, vs 142/518, 27.4%; RR 0.99, 95% CI 0.97-1.02; *P*=.65). The intervention measures improved smoking cessation (44/565, 7.8%, vs 118/518, 22.8%; RR 0.48, 95% CI 0.44-0.53; *P*<.001) and alcohol restriction (33/565, 5.8%, vs 91/518, 17.6%; RR 0.47, 95% CI 0.42-0.54; *P*<.001).

**Conclusions:**

The tertiary A-level hospital, WeChat-based intervention did not improve adherence to the 4 cardioprotective medications compared with the traditional method. Tertiary A-level hospital, WeChat-based interventions have a positive effect on improving lifestyle, such as quitting drinking and smoking, in patients with stable coronary artery disease and can be tried as a supplement to community hospital follow-up.

**Trial Registration:**

ClinicalTrials.gov NCT04795505; https://clinicaltrials.gov/ct2/show/NCT04795505

## Introduction

### Background

In China, the main cause of mortality is a cardiac condition known as ischemic heart disease [1]. According to current recommendations, having cardiac rehabilitation and a secondary prevention program in place is a class IA recommendation for individuals with coronary artery disease (CAD) [2-4]; however, there is a large gap between clinical practice and guideline recommendations. The cost of treating cardio-cerebrovascular illnesses in China was Chinese ¥540.64 billion (US $83.90 billion) in 2017. More than 80% of the costs of cardio-cerebrovascular diseases in China were incurred in hospitals and over 70% of the costs incurred in inpatient care. These allocations were unreasonable, and the primary medical and health facilities accounted for less than 12% of the costs [5]. To reduce the economic burden of cardiovascular illnesses in China, efforts have concentrated on improving the quality of treatment for acute myocardial infarctions (MIs) and percutaneous coronary interventions (PCIs) [6]. Since the development of a clinical performance quality control system for adults with acute ST-elevation MI, significant improvements have been achieved in China regarding the prescription of medications during hospitalization, and these medications are evidence-based [7]. However, approximately half of the patients with acute MI in China do not have good compliance in taking their medications after discharge, which substantially increases morbidity and mortality [8-10]. It is difficult for patients to be hospitalized in tertiary A-level hospitals in China, and many patients do not return for follow-up after discharge due to the patient perspective of treatment being much more important than prevention. The low-density lipoprotein cholesterol (LDL-C) goals were not met by a statistically significant percentage (74.5%) of individuals with a high risk of arteriosclerotic cardiovascular disease [11]. First- and second-level preventive care needs to be improved to increase patient compliance and to change modifiable risk factors [12,13].

In response to this phenomenon, facilities and agencies are trying to engage patients, change behaviors, and help to control the risk factors. Traditional patient education methods include in-office patient counseling, health seminars, follow-up via telephone calls, text messages or emails, etc. Traditional teaching methods had no effect on fatal or nonfatal MI, total revascularization, or hospitalization, according to a Cochrane comprehensive study [14], and innovative strategies are required for routine clinical use.

Tencent introduced WeChat (Chinese version: Wei Xin), a free social networking app, in January 21, 2011, to offer instant messaging services across all platforms. It not only offers basic text, voice, photo and video sharing, web-based payment, and news subscription services but also provides integration with intelligent hardware, such as smart bracelets, blood pressure (BP) monitors, and body fat scales. WeChat now has over one billion active users, making it the most popular social networking site on the planet. After considering its extensive population coverage, strong peripheral features, and seamless integration into everyday life [15], many hospitals have introduced web-based follow-up measures based on WeChat to strengthen secondary prevention measures and risk factor interventions and to improve the drug compliance of patients. WeChat-based interventions seem to be feasible and efficient for the follow-up and management of chronic diseases. A review by Chen et al [16] discussed the following reasons why WeChat might be useful in chronic illness management: (1) it provides continuous health services. Hospitals or community health centers might develop distinct WeChat groups or official WeChat accounts based on the categories of chronic illnesses. (2) WeChat can help patients change their unhealthy lifestyle by constantly sending patient education materials to them. (3) A WeChat-based follow-up approach can improve physician-patient relationships by delivering personalized health advice and enhancing user engagement. (4) Doctors can spread their successful experiences and measures widely and quickly to many patients through group messages.

### Objectives

To our knowledge, no studies have compared WeChat web-based interventions with traditional community hospital follow-ups [17,18] This study evaluates the benefits of a tertiary A-level hospital WeChat-based telemedicine in comparison with a conventional community hospital follow-up on medication adherence and risk factor control in individuals with stable CAD.

## Methods

### Study Design

A secondary prevention telemedicine program based on the WeChat platform provided by a tertiary A-level hospital was assessed in this 2-arm, parallel multicenter prospective study. It was one of the Prevention and Control Projects of the Major Chronic Noninfectious Disease (grant 2018YFC1315600), which was supported by the Ministry of Science and Technology of China. The National Center for Cardiovascular Diseases and the Fuwai Hospital led the study design, follow-up, data collection, and analysis of this study. Trial development and reporting were in accordance with the Strengthening the Reporting of Observational Studies in Epidemiology Statement. We registered this study on ClinicalTrials.gov (NCT04795505).

At the initial trial visit, all participants signed a written informed consent form, and the study adhered to the principles of the Declaration of Helsinki. The primary ethical committee of the National Center for Cardiovascular Diseases approved the research protocol.

### Recruitment

In this multicenter prospective study, 1424 patients with stable CAD in Beijing, China, were consecutively enrolled between September 2018 and September 2019 from the Fuwai Hospital and 4 community hospitals. The inclusion criteria were as follows: participants were required to be aged at least 18 years and to have a diagnosis of stable CAD according to the guidelines [19,20]. All participants underwent coronary computed tomography angiography or coronary angiography. Patients who could potentially participate in the research were checked as outpatients and given a form to return with their information. Participants in the intervention group were required to own a smartphone with an active WeChat account and to have the ability to communicate fluently in Chinese with the cardiac rehabilitation team via WeChat. Participants in the control group were eligible to participate if they were registered in one of the 4 community hospitals. Participants were excluded if they refused to provide signed informed consent or had a life expectancy of less than a year because of comorbidities. Participants were assigned to either the intervention or control group at their own discretion. Demographic information and reasons for study withdrawal were recorded for each participant during the entire study period.

### Interventions

**Figure 1:**
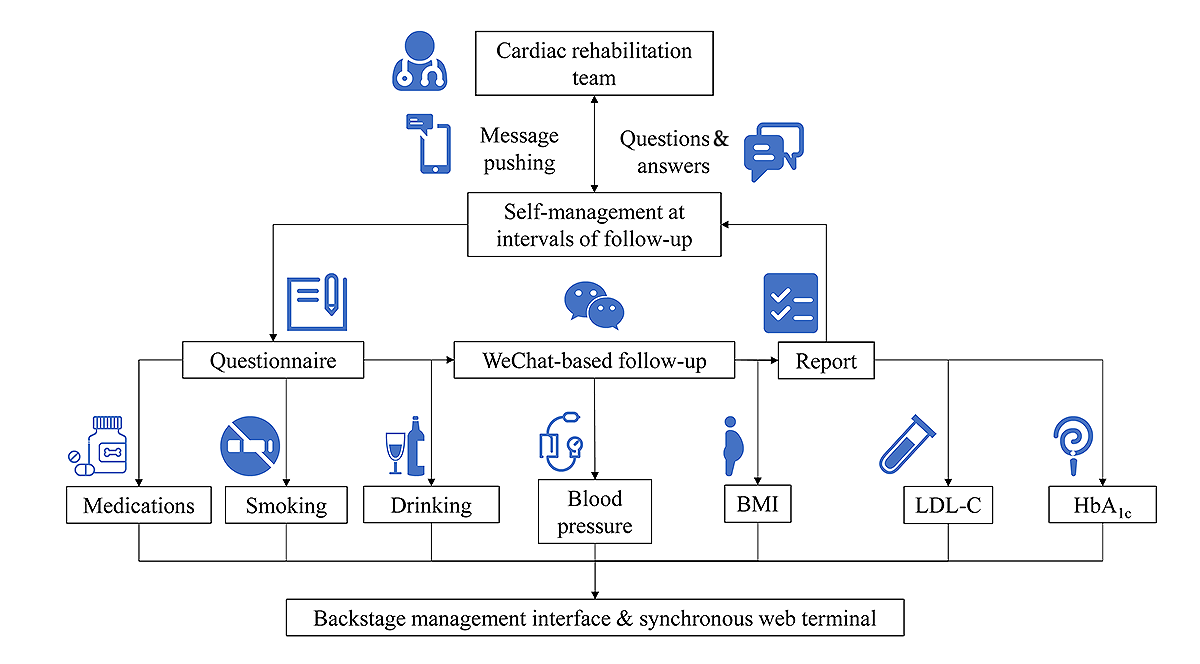
Overview of the WeChat-based telemedicine intervention. HbA_1c_: glycated hemoglobin A_1c_; LDL-C: low-density lipoprotein cholesterol.

**Figure 2:**
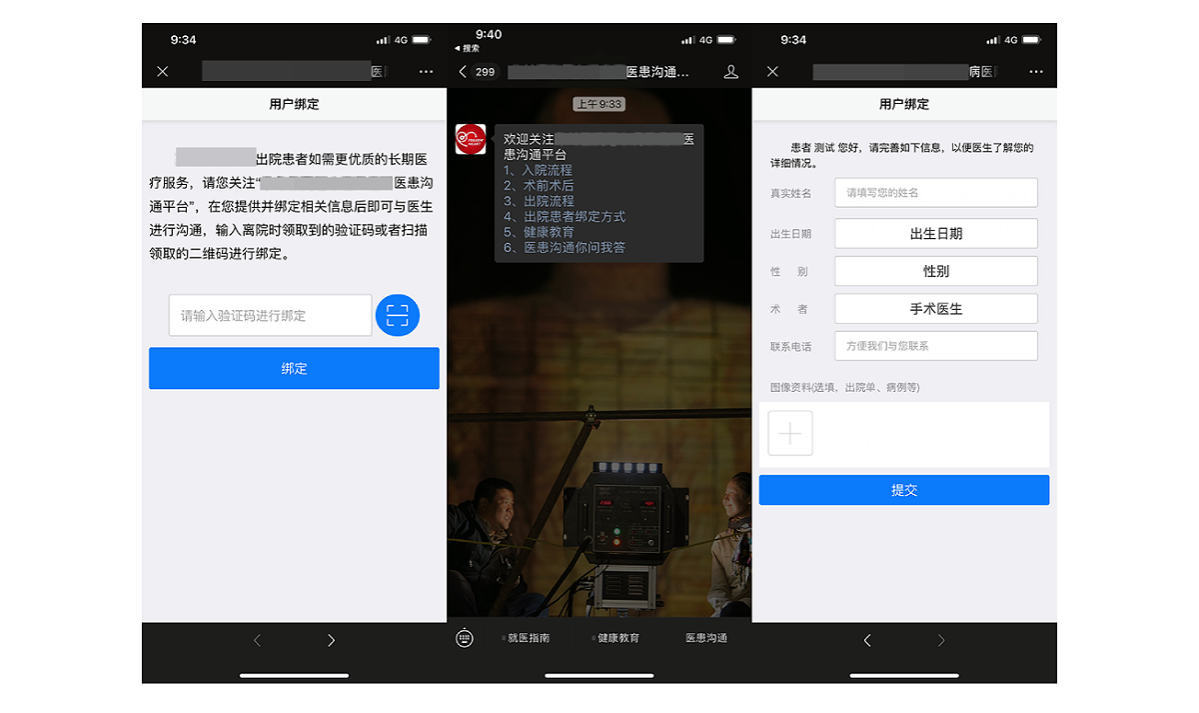
Screenshots of the user registration and binding interface.

**Figure 3:**
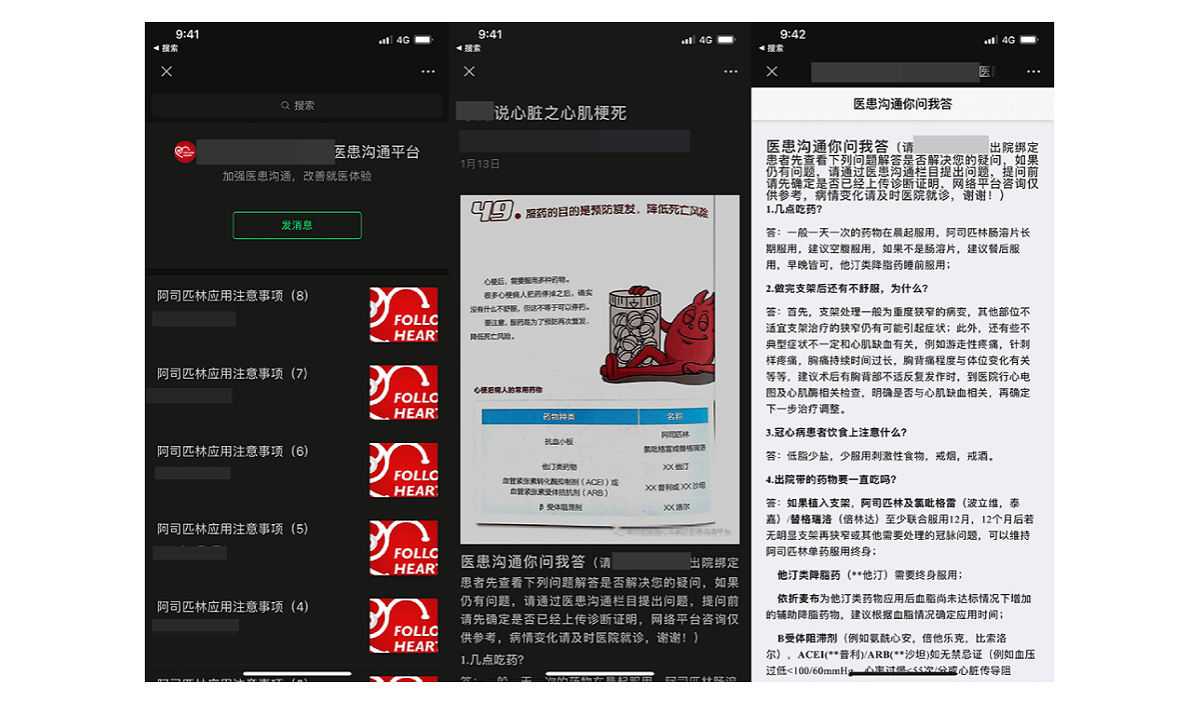
Screenshots of educational materials related to coronary heart disease in the patient terminal.

**Figure 4:**
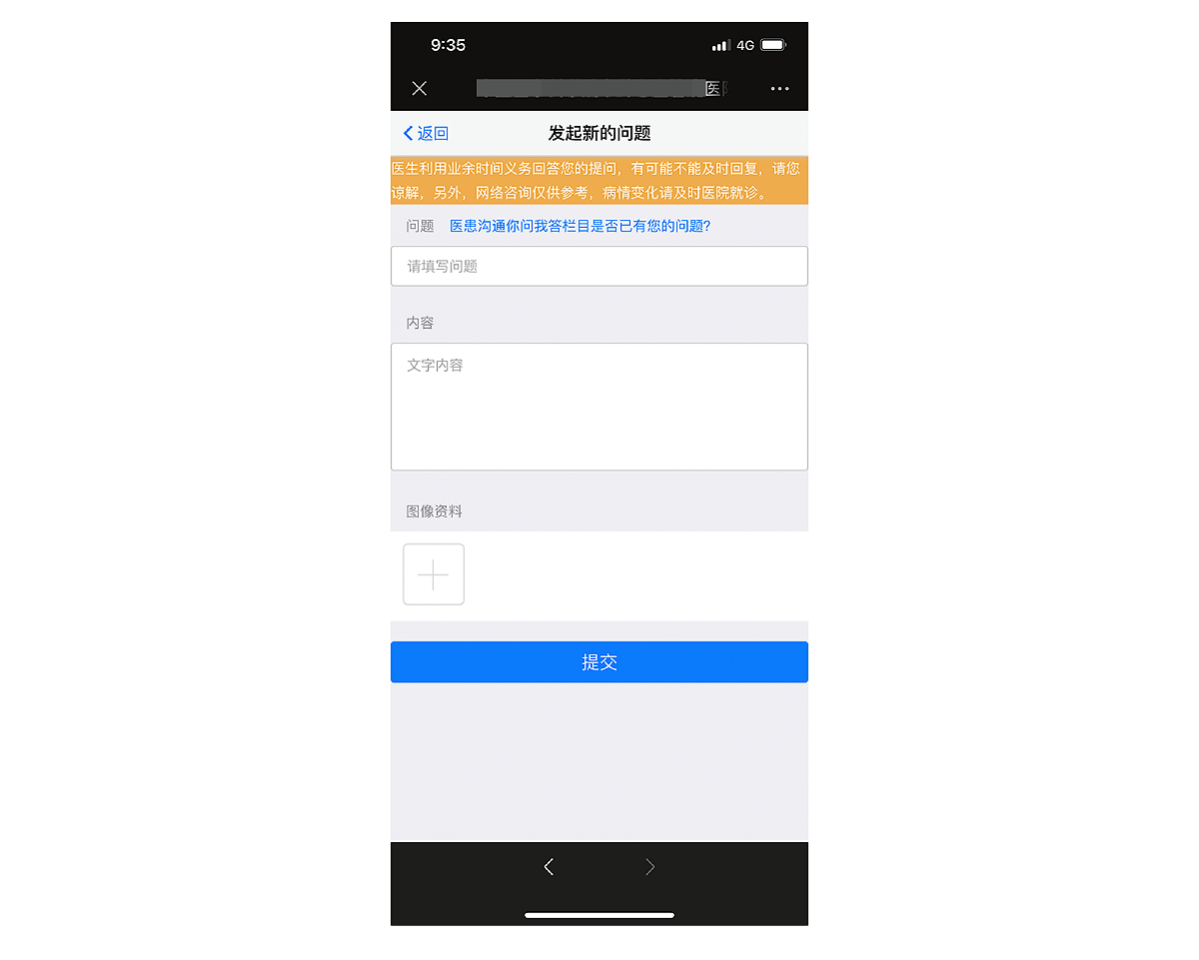
Screenshot of the initiation of a question in the patient terminal.

**Figure 5:**
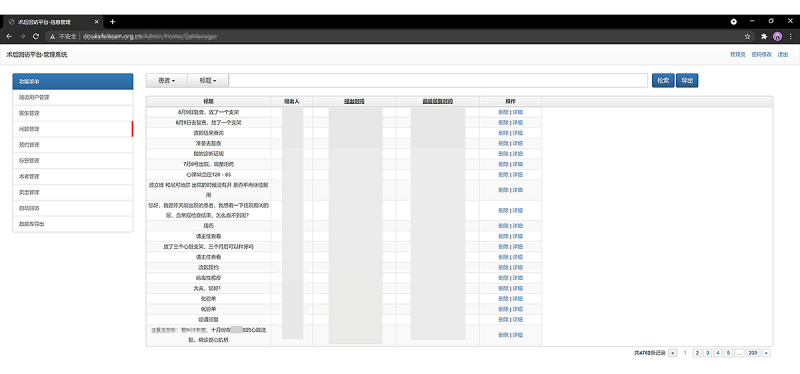
Backstage management interface of the WeChat-based secondary prevention program.

**Figure 6:**
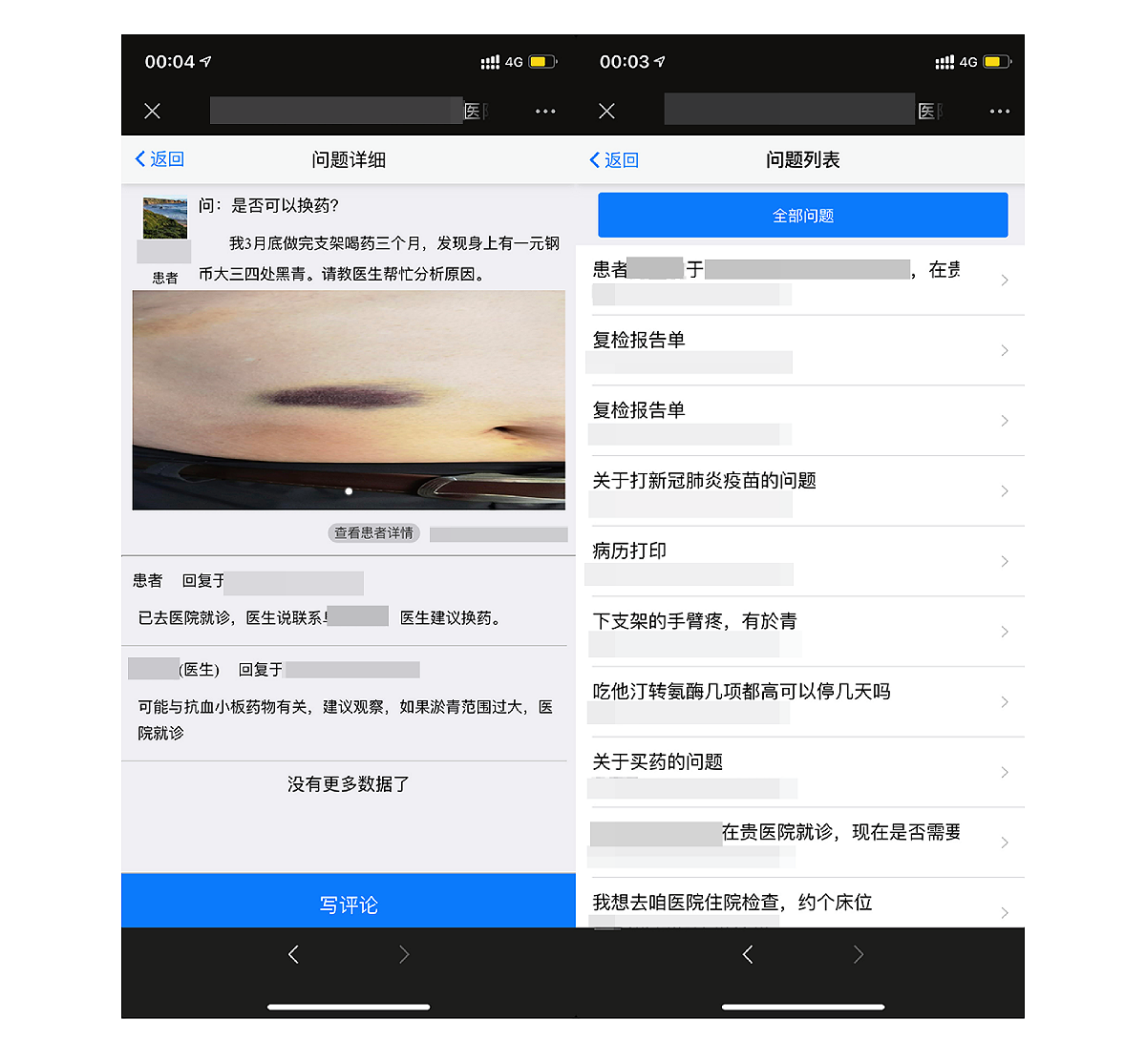
Screenshots of the answering of questions in the physician terminal.

At the 1-, 3-, 6-, and 12-month follow-ups, participants received healthy lifestyle recommendations and medication advice. Subsequently, the control group went to an offline outpatient clinic at 4 separate community hospitals. The control group received conventional outpatient cardiology care, including formal cardiac rehabilitation and secondary preventive measures whereas the intervention group had follow-up visits through WeChat-based telemedicine management (Figure 1). Participants in this group were trained on how to interact with the WeChat official account (Figure 2). Each appointment included inquiries, evaluations, and comments. A questionnaire (Multimedia Appendix 1) was administered remotely before formal WeChat-based follow-up. The questionnaire included symptoms and adverse events, control of risk factors, basic physical examination and auxiliary examination, and medication status. Participants can answer the above questions by voice, text, or picture. The results of the questionnaire are only for improving the efficiency of information collection, and doctors will further confirm the authenticity of information during follow-up visits on WeChat. On the basis of the above preliminary data, the researcher appointed time to further communicate with the subjects on WeChat and took intervention measures such as adjusting the treatment plan, strengthening the control of risk factors, and improving the lifestyle. During every consultation, the participant’s medication adherence and risk factor modification status were evaluated, and the participant was given personalized feedback, encouragement, and suggestions. In our study, risk factor modification included improving cholesterol management, quitting smoking and drinking, monitoring BP, and maintaining a healthy weight. At the end of each visit, the participant received an evaluation report (Multimedia Appendix 2), highlighting areas for improvement. The researchers focused on outcomes where the participant did not perform well at the previous follow-up to trigger a virtuous circle and help them achieve optimal cardiovascular health. The official WeChat account also had other functions, such as regularly sending health education materials, physician-patient communication, and medical appointments. Participants were provided with a variety of teaching materials on coronary heart disease that had been evaluated by cardiologists and that they may read whenever and wherever they wished (Figure 3). Relevant information was updated and sent weekly. If participants had questions, they could always ask the physician in the form of text and pictures via WeChat (Figure 4). Doctors could see participants’ questions in the backstage (Figure 5) and answer them on a mobile phone (Figure 6). Participants would be contacted by phone call if their condition changed or they cannot be contacted by WeChat, or the investigator deemed it necessary. The cardiac rehabilitation team received uniform training before first contact with the participant to minimize the heterogeneity of the interventions.

### Outcome Measures and Data Collection

Participant characteristics included age, gender, alcohol consumption, and cigarette smoking, clinical data (systolic BP, diastolic BP, BMI, LDL-C, glycated hemoglobin A_1c_ [HbA_1c_], and ejection fraction), past medical history (hypertension, dyslipidemia, CAD family history, previous PCI, previous coronary artery bypass grafting, previous MI, previous ischemic stroke, diabetes mellitus [DM], peripheral vascular disease, chronic kidney disease, and heart failure), and medications (antiplatelets, β-blockers, statins, and angiotensin-converting-enzyme inhibitor or angiotensin-receptor blocker [ACEI/ARB]).

The main endpoint was medication adherence, which was defined as the participants compliance in taking all 4 cardioprotective medications that would improve their outcome (therapies included antiplatelet therapy, β-blockers, statins, and ACEI/ARB). Participants were considered to be taking all 4 of the cardiovascular protective medications if they were taking all 4 medications at the time of the follow-up and had no more than 10% of the days without medication. If the participant can provide details of the prescription, the investigator will calculate the medication status based on the prescription. The secondary outcomes included control of hypertension, current smoking, current alcohol consumption, 18.5≤BMI<25.0 kg/m^2^, LDL-C<1.8 mmol/L and HbA_1c_<7%. BP less than 140/90 mm Hg in individuals was considered to have good control of hypertension for the purposes of this research. These target values are based on guidelines for the diagnosis and treatment of stable CAD [20]. The whole data set included 4 parts: baseline characteristics and the 1-, 3-, 6-, and 1-year follow-up characteristics. All the baseline characteristics were extracted from the participants’ medical records.

Researchers performed face-to-face interviews with participants in the control group during the first visit, as well as at the 1-month, 3-month, 6-month, and 1-year follow-up visits. At the follow-up, body height and body weight were measured by the physicians. Two BP readings were taken by using an electronic BP monitor with the participant sitting in a chair with back support after 10 minutes of rest, and the average was considered as the final reading. Behavioral changes in drinking and smoking status and adherence to secondary prevention medications were self-reported by the participants. We evaluated 4 medications that were commonly prescribed to patients with stable CAD; specifically, each participant was asked about their current medications during each follow-up visit.

Follow-up data were collected for the intervention group via our official WeChat account. To minimize the impact of the discrepancy between BP recorded at home and BP measured at the clinic, all members of this group were requested to report their height, weight, and BP using conventional procedures at a nearby clinic. Behavioral changes in drinking and smoking status and adherence to medications were collected using self-reported questionnaires. Blood samples in both groups for LDL-C and HbA_1c_ levels were analyzed in the respective laboratories using standard procedures.

**Figure 7:**
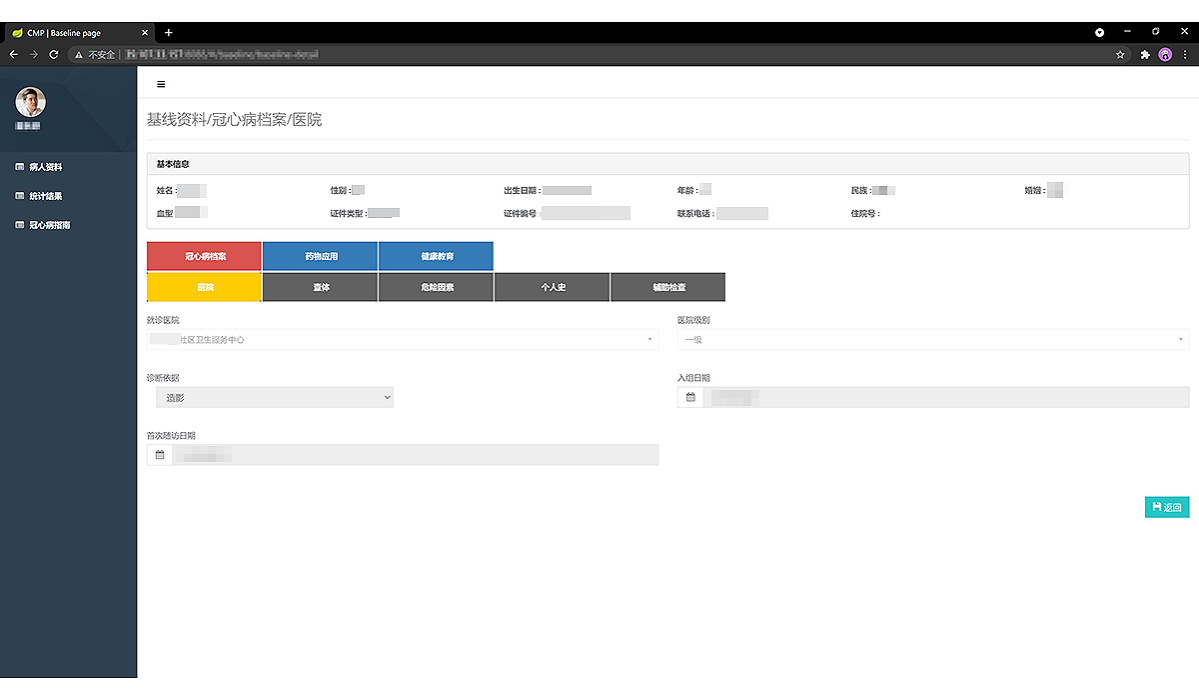
Synchronous data-capture system of the WeChat-based secondary prevention program.

An electronic data capture system (Figure 7) was used to gather and handle all the data. To enter and analyze the data, researchers needed to be given appropriate permissions, and all the researchers were unable to access the database until they underwent data safety training.

### Statistical Analysis

Categorical variables were described using frequencies and percentages, and continuous variables using means with SDs or medians with IQRs. The baseline characteristics of participants were compared across groups using the chi-square or Fisher exact test for categorical variables and Student *t* tests (2-tailed) or the Wilcoxon rank-sum test for continuous data. Multivariable generalized estimating equations (GEEs) were used to compare the primary and secondary outcomes between the 2 groups and to calculate the relative risk (RR) at 12 months. Propensity score matching (PSM) and inverse probability of treatment weighting (IPTW) were performed as sensitivity analyses, and propensity scores were calculated using a multivariable logistic regression model. These variables, including gender, age, current smoking, current alcohol consumption, hypertension, dyslipidemia, CAD family history, previous coronary artery bypass grafting, previous PCI, previous MI, ischemic stroke, DM, peripheral vascular disease, chronic kidney disease, heart failure, BP, BMI, LDL-C, HbA_1c_, antiplatelet medications, β-blocker use, statin use, and ACEI/ARB, were chosen as covariates because the differences in the baseline characteristics reached statistical significance (*P*<.10) or were associated with the outcome. The PSM process was based on the nearest neighbor matching algorithm without replacement under a 0.02 caliper at a 1:1 ratio, yielding 257 participants in the intervention group and 257 participants in the control group. IPTW was performed using the same propensity score as previously estimated. A standardized mean difference of <0.2 indicated an acceptable balance after matching or weighting. We used this set of tests to account for baseline variables and draw conclusions about the effect of telemedicine intervention on the results at the individual participant level. Furthermore, comparisons of the primary endpoint between the 2 groups were made based on the prespecified baseline characteristics including gender, age, control of hypertension, current smoker, current drinker, BMI, LDL-C, and HbA_1c_ subgroups. The interaction between treatment effects and subgroups was evaluated using the multivariable GEE models. The analysis was performed in the whole population and adjusted for baseline factors including gender, age, control of hypertension, current smoker, current drinker, BMI, LDL-C, and HbA_1c_. On the basis of previous studies [8,18], it is estimated that the proportion of the control group in this study who were persistent with taking the 4 cardiovascular protective drugs at the 1-year follow-up was approximately 30%, whereas the proportion of the intervention group was estimated to be 40%. We calculated according to a 90% power (2-sided α=.05) and considering a 10% participant loss to follow-up, a total of 1060 participants needed to be enrolled in this study. The ratio between the intervention and control groups was 1:1, and 530 participants were included in the 2 groups.

A 2-tailed *P* value <.05 was considered statistically significant. All the statistical analyses were performed using STATA 16.0 (Stata Corp) and R 4.0.2 (R Foundation for Statistical Computing). The missing values are filled in by the average of the 10 multiple interpolations. None of the variables had missing values of >5%. Missing values varied from 0.1% (BP) to 3.6% (HbA_1c_).

## Results

### Baseline Characteristics

Table 1 summarizes the unadjusted baseline characteristics.

**Table 1:** Baseline characteristics (N=1206).

Variables		Total	Intervention (n=642)	Control (n=564)	*P* value	SMD^a^
**Demographics**						
	Male, n (%)	875 (72.6)	488 (76)	387 (68.6)	.005	0.166
	Age (years), mean (SD)	64.83 (10.59)	61.27 (10.20)	68.89 (9.52)	<.001	0.772
	Age ≥65 years, n (%)	637 (52.8)	253 (39.4)	384 (68.1)	<.001	0.601
	Current smoker, n (%)	308 (25.5)	171 (26.6)	137 (24.3)	.39	0.054
	Current drinker, n (%)	192 (15.9)	93 (14.5)	99 (17.6)	.17	0.084
**Past medical history, n (%)**						
	Hypertension	856 (71)	409 (63.7)	447 (79.3)	<.001	0.35
	Dyslipidemia	907 (75.2)	564 (87.9)	343 (60.8)	<.001	0.651
	CAD^b^ family history	120 (10)	60 (9.3)	60 (10.6)	.52	0.043
	Previous CABG^c^	59 (4.9)	20 (3.1)	39 (6.9)	.004	0.175
	Previous PCI^d^	304 (25.2)	205 (31.9)	99 (17.6)	<.001	0.338
	Previous myocardial infarction	172 (14.3)	99 (15.4)	73 (12.9)	.25	0.071
	Previous ischemic stroke	85 (7)	53 (8.3)	32 (5.7)	.10	0.102
	Diabetes mellitus	464 (38.5)	205 (31.9)	259 (45.9)	<.001	0.29
	Peripheral vascular disease	40 (3.3)	26 (4)	14 (2.5)	.18	0.088
	Chronic kidney disease	11 (0.9)	2 (0.3)	9 (1.6)	.04	0.132
	Heart failure	34 (2.8)	30 (4.7)	4 (0.7)	<.001	0.247
**Clinical data**						
	Good control of hypertension, n (%)	860 (71.3)	369 (57.5)	491 (87.1)	<.001	0.7
	Systolic BP^e^ (mm Hg), mean (SD)	131.65 (16.33)	135.10 (18.67)	127.73 (12.04)	<.001	0.47
	Diastolic BP (mm Hg), mean (SD)	77.06 (10.38)	78.06 (11.92)	75.93 (8.16)	<.001	0.209
	BMI (kg/m^2^), mean (SD)	25.61 (3.19)	25.74 (3.21)	25.45 (3.17)	.12	0.091
	18.5≤BMI<25.0 kg/m^2^, n (%)	516 (42.8)	276 (43)	240 (42.6)	.92	0.009
	LDL-C^f^ (mmol/L), mean (SD)	2.32 (0.80)	2.34 (0.84)	2.29 (0.75)	.34	0.055
	LDL-C<1.8 mmol/L, n (%)	328 (27.2)	172 (26.8)	156 (27.7)	.79	0.02
	HbA_1c_^g^ (%), mean (SD)	6.50 (1.18)	6.55 (1.23)	6.44 (1.11)	.11	0.094
	HbA_1c_<7%, n (%)	903 (74.9)	475 (74)	428 (75.9)	.49	0.044
**Medications, n (%)**						
	Medications adherence	374 (31)	211 (32.9)	163 (28.9)	.16	0.086
	Antiplatelet	1182 (98)	632 (98.4)	550 (97.5)	.35	0.066
	β-blocker	867 (71.9)	514 (80.1)	353 (62.6)	<.001	0.394
	Statin	1144 (94.9)	633 (98.6)	511 (90.6)	<.001	0.359
	ACEI/ARB^h^	565 (46.8)	277 (43.1)	288 (51.1)	.007	0.159

^a^SMD: standardized mean difference.

^b^CAD: coronary artery disease.

^c^CABG: coronary artery bypass grafting.

^d^PCI: percutaneous coronary intervention.

^e^BP: blood pressure.

^f^LDL-C: low-density lipoprotein cholesterol.

^g^HbA_1c_: glycated hemoglobin A_1c_.

^h^ACEI/ARB: angiotensin-converting-enzyme inhibitor or angiotensin-receptor blocker.

**Figure 8:**
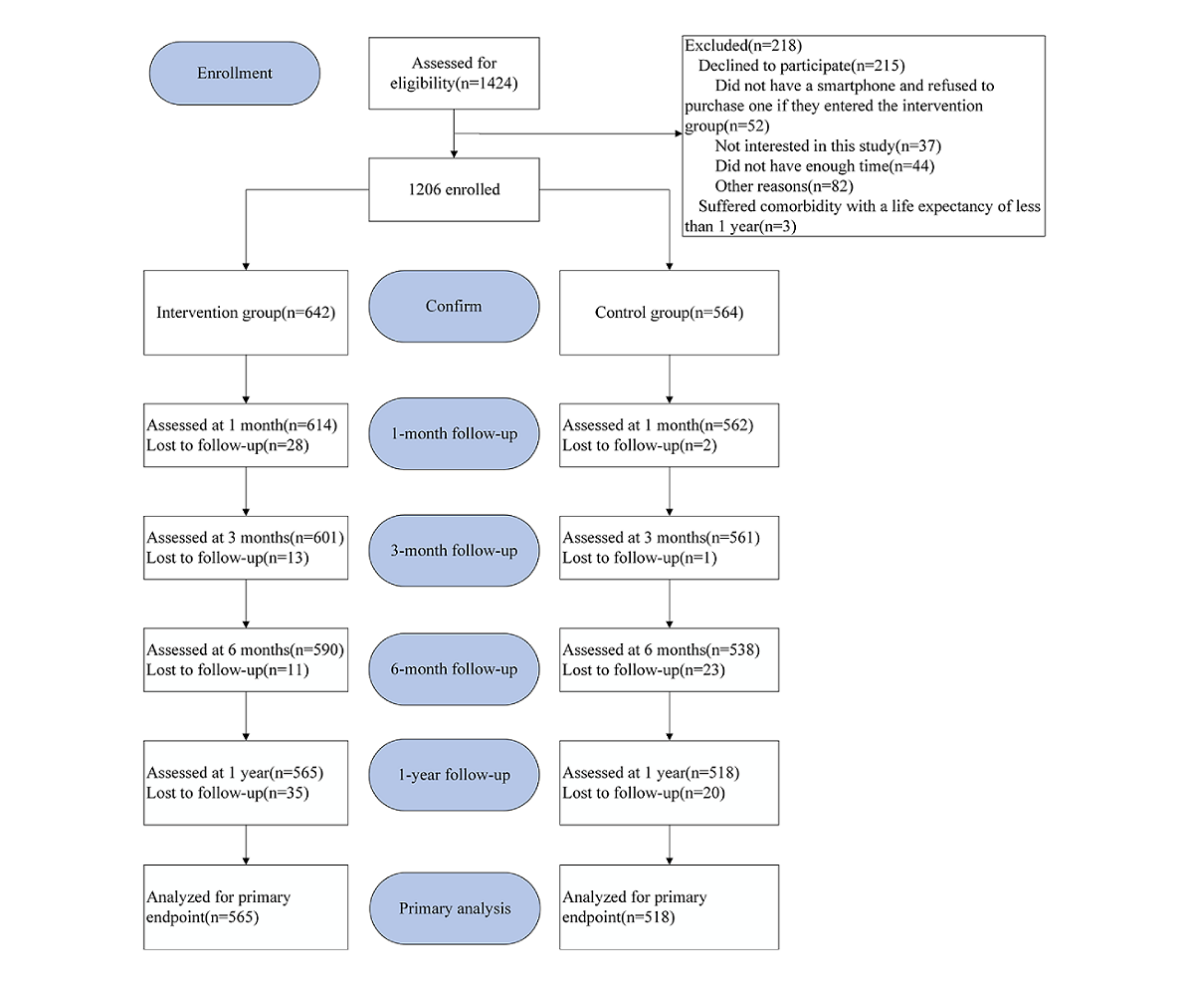
Participant flow diagram.

In this study, 1424 participants were identified between September 2018 and September 2019. After screening the participants based on the exclusion criteria, 1206 participants were analyzed in this study. At 1 year, 88% (565/642) of participants in the intervention group and 91.8% (518/564) of participants in the control group had successful follow-up data (Figure 8). The loss to follow-up rate was lower in the control group (77/642, 12%, vs 46/564, 8.2%; *P*=.03), and 84.1% (475/565) of participants in the intervention group were followed up via the WeChat platform. In summary, participants in the intervention group were more likely to be male (488/642, 76%, vs 387/564, 68.6%; *P*<.001) and younger (61.27 vs 68.89; *P*<.001). The intervention group showed a reduced prevalence of comorbidities such as hypertension (409/642, 63.7%, vs 447/564, 79.3%; *P*<.001), DM (205/642, 31.9%, vs 259/564, 45.9%; *P*<.001), and chronic kidney disease (2/642, 0.3%, vs 9/564, 1.6%; *P*<.001) when compared with the control group. Regarding clinical data, the intervention group had worse BP control (369/642, 57.5%, vs 491/564, 87.1%; *P*<.001) than the control group; however, heart failure was more common in the intervention group (30/642, 4.7%, vs 4/564, 0.7%; *P*<.001) as was dyslipidemia (564/642, 87.9%, vs 343/564, 60.8%; *P*<.001). Regarding medication adherence with the 4 cardioprotective drugs, participants in the intervention group more frequently received β-blockers (514/642, 80.1%, vs 163/564, 62.6%; *P*<.001) and statins (633/642, 98.6%, vs 163/564, 90.6%; *P*<.001) and less frequently received ACEI/ARB (277/642, 43.1%, vs 288/564, 51.1%; *P*=.007). There were no statistically significant differences between the 2 groups with regard to current smoker, current drinker, previous PCI, previous MI, previous ischemic stroke, medication adherence, BMI, LDL-C, or HbA_1c_. Overall, the on-target proportions of BP, BMI, LDL-C, and HbA_1c_ were 71.31% (860/1206), 42.79% (516/1206), 27.2% (328/1206), and 74.88% (903/1206), respectively, and 54.7% (254/464) of patients with known diabetes had HbA_1c_≥7%. Regarding unhealthy lifestyles, the proportions of smokers and drinkers were 25.54% (308/1206) and 15.92% (192/1206), respectively. The prevalence of the 4 cardiovascular drugs at the beginning was 31.01% (374/1206, 95% CI 28.4%-33.6%). Among them, the proportion of antiplatelet drugs (98.01%, 1182/1206) and statins (94.86%, 1144/1206) was higher, whereas the proportion of β-blockers (71.89%, 867/1206) and ACEI/ARBs (46.85%, 565/1206) was lower. Among participants treated with statins, 73.6% (842/1144) did not achieve the goal LDL-C level of 1.8 mmol/L. The reasons for participants’ loss of follow-up included not being able to keep in touch (82.1%, 101/123) and participants requesting withdrawal from the study (17.9%, 22/123). Clinical demographics of follow-up and lost to follow-up participants are shown in Multimedia Appendix 3. Compared with participants with regular follow-up, participants who were lost to follow-up had a higher proportion of hypertension (757/1083, 69.9%, vs 99/123, 80.5%; *P*=.02), diabetes (403/1083, 37.21%, vs 61/123, 49.6%; *P*=.01), a lower proportion of dyslipidemia (825/1083, 76.18%, vs 82/123, 66.7%; *P*=.03), and better medication adherence (324/1083, 29.92%, vs 50/123, 40.7%; *P*=.02).

### Sensitivity Analyses Using PSM and IPTW

We matched 257 pairs of participants between the intervention group and the control group using PSM. To avoid decreasing the sample size and weakening the statistical power, we also performed IPTW using the same covariates in the PSM. After matching and weighing, almost all covariates were well-balanced, except for age (Multimedia Appendix 4). Detailed baseline characteristics and standard mean differences after PSM and IPTW are depicted in Multimedia Appendix 5.

### Primary and Secondary Outcome Analyses

Table 2 presents an overview of the primary and secondary outcomes at the 1-year follow-up (comparison within groups).

**Table 2:** Primary and secondary outcomes at the 1-year follow-up (comparison within groups).

Outcomes			Intervention							Control			
			Baseline, n (%)		1 year, n (%)		*P* value		Baseline, n (%)			1 year, n (%)	*P* value
**Primary outcome**													
	Medication adherence	211 (32.9)		172 (30.4)		.38		163 (28.9)			142 (27.4)		.63
**Secondary outcomes**													
	Antiplatelet	632 (98.4)		540 (95.6)		.005		550 (97.5)			494 (95.4)		.08
	β-blocker	514 (80.1)		441 (78.1)		.42		353 (62.6)			329 (63.5)		.80
	Statin	633 (98.6)		532 (94.2)		<.001		511 (90.6)			479 (92.5)		.32
	ACEI/ARB^a^	277 (43.1)		227 (40.2)		.31		288 (51.1)			258 (49.8)		.73
	Current smoker	171 (26.6)		44 (7.8)		<.001		137 (24.3)			118 (22.8)		.61
	Current drinker	93 (14.5)		33 (5.8)		<.001		99 (17.6)			91 (17.6)		.99
	Good control of hypertension	369 (57.5)		416 (73.6)		<.001		491 (87.1)			486 (93.8)		<.001
	18.5≤BMI<25.0 kg/m^2^	276 (43)		237 (41.9)		.74		240 (42.6)			226 (43.6)		.77
	LDL-C^b^<1.8 mmol/L	172 (26.8)		198 (35)		.002		156 (27.7)			280 (54.1)		<.001
	HbA_1c_^c^<7%	475 (74)		439 (77.7)		.16		428 (75.9)			484 (93.4)		<.001

^a^ACEI/ARB: angiotensin-converting-enzyme inhibitor or angiotensin-receptor blocker.

^b^LDL-C: low-density lipoprotein cholesterol.

^c^HbA_1c_: glycated hemoglobin A_1c_.

Compared with the previous year, there was no significant difference in the drug adherence with the 4 cardioprotective medications in either the intervention or the control group (172/565, 30.4%, vs 211/642, 32.9%, *P*=.38; 142/518, 27.4%, vs 163/564, 28.9%, *P*=.63). Compared with the previous year, an increased prevalence of good hypertension management was observed among the intervention group (416/565, 73.6%, vs 369/642, 57.5%; *P*<.001), an LDL-C on target (198/565, 35%, vs 172/642, 26.8%; *P*<.001) and a reduction in the proportion of current smokers (44/565, 7.8%, vs 171/642, 26.6%; *P*<.001) and drinkers (33/565, 5.8%, vs 93/642, 14.5%; *P*<.001). After the 1-year follow-up, the proportion of medication adherence to antiplatelet treatment (540/565, 95.6%, vs 632/642, 98.4%; *P*=.005) and statins (532/565, 94.2%, vs 633/642, 98.6%; *P*<.001) decreased. In the control group, participants achieved a better BP level (486/518, 93.8%, vs 491/564, 87.1%; *P*<.001), improved lipid levels (280/518, 54.1%, vs 156/564, 27.7%; *P*<.001) and improved control of blood glucose (484/518, 93.4%, vs 428/564, 75.9%; *P*<.001) at the 1-year follow-up.

**Figure 9:**
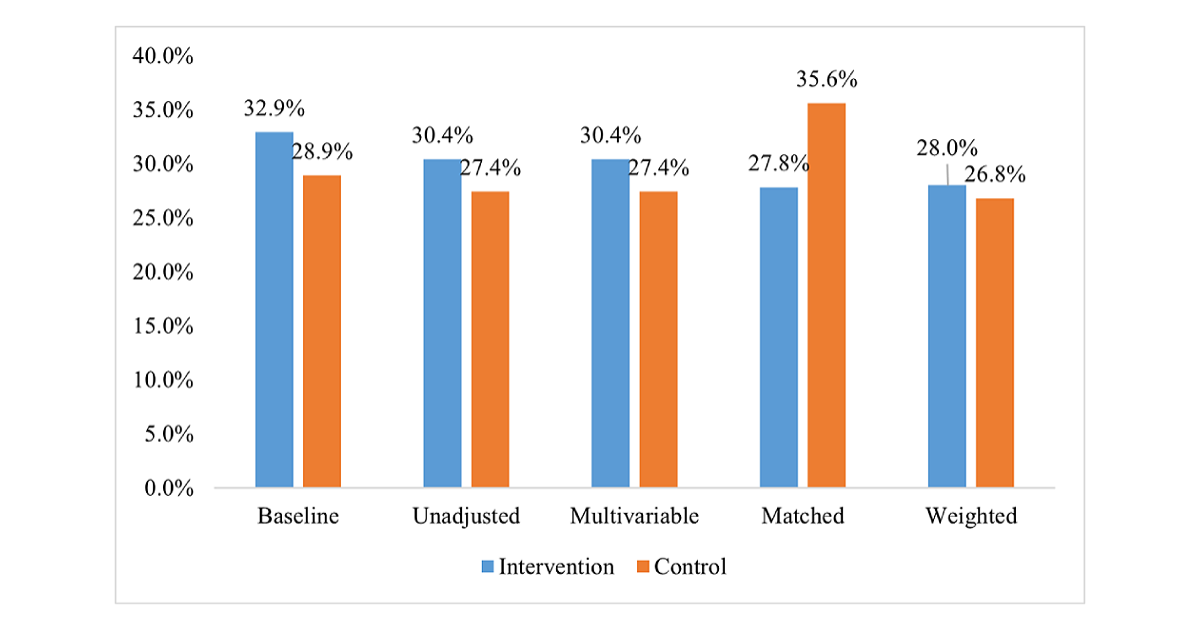
Proportions of medical adherence to 4 cardioprotective drugs in the intervention group and control group.

Multimedia Appendix 6 presents 1-year primary and secondary outcomes (intervention vs control). Figure 9 depicts proportions of medical adherence to the 4 cardioprotective drugs in the intervention group and control group by different statistical methods. Compared with the routine follow-up in community hospitals, there was no obvious advantage in the medication adherence with the 4 cardioprotective drugs in the intervention group (172/565, 30.4%, vs 142/518, 27.4%; RR 0.99, 95% CI 0.97-1.02; *P*=.65). The mean difference of medications adherence between the intervention and control groups is 3% (95% CI 0.2%-11.5%). The intervention measures improved the smoking cessation (44/565, 7.8%, vs 118/518, 22.8%; RR 0.48, 95% CI 0.44-0.53; *P*<.001), alcohol restriction (33/565, 5.8%, vs 91/518, 17.6%; RR 0.47, 95% CI 0.42-0.54; *P*<.001). The control group was superior to the intervention group in medication adherence in regard to ACEI/ARBs (227/565, 40.2%, vs 258/518, 49.8%; RR 0.98, 95% CI 0.96-0.99; *P*<.001), BMI (237/565, 41.9%, vs 226/518, 43.6%; RR 0.95, 95% CI 0.93-0.97; *P*<.001), LDL-C (198/565, 35%, vs 280/518, 54.1%; RR 0.79, 95% CI 0.73-0.84; *P*<.001), and blood glucose (439/565, 77.7%, vs 484/518, 93.4%; RR 0.95, 95% CI 0.94-0.97; *P*<.001) targets. All of these results were still significant after the multivariable analysis using GEE, PSM, and IPTW.

### Subgroup Analysis

**Figure 10:**
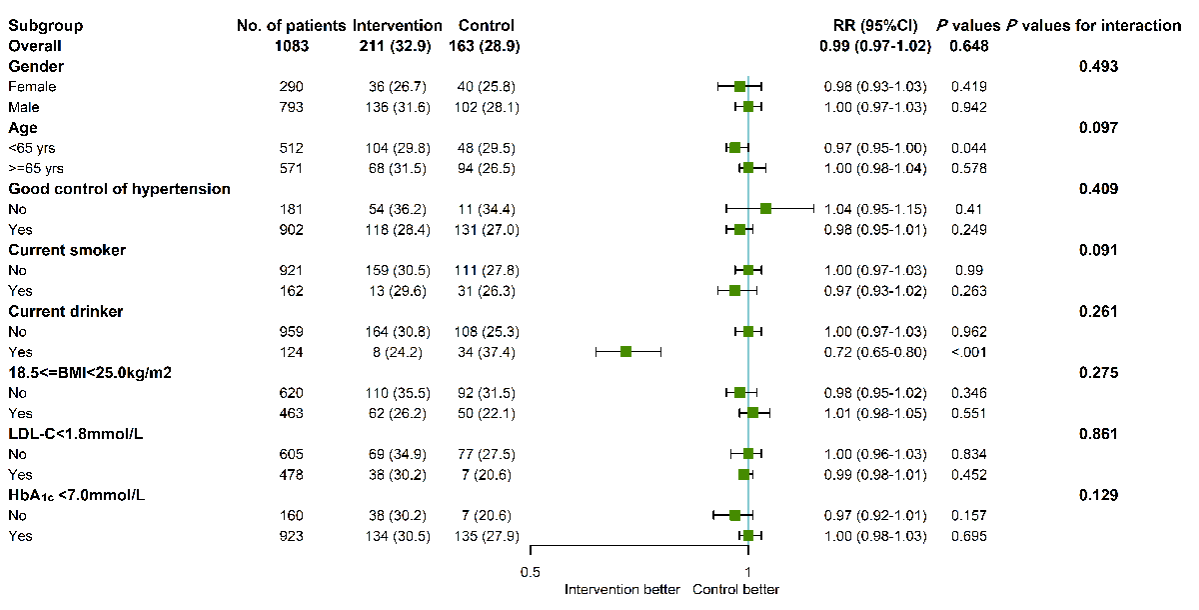
Subgroup analysis of primary outcome. Values are n (%) for categorical variables. The interaction between treatment effect and subgroups was evaluated using multivariable generalized estimating equations models. The analysis was performed in the whole population and adjusted for baseline factors including sex, age, control of hypertension, current smoker, current drinker, BMI, low-density lipoprotein cholesterol, and glycated hemoglobin. HbA_1c_: glycated hemoglobin A_1c_; LDL-C: low-density lipoprotein cholesterol. RR: relative risk.

The intervention and control groups were divided into 16 subgroups according to gender, age, control of hypertension, current smoker, current drinker, BMI, LDL-C, and HbA_1c_ (Figure 10). No significant difference in medication adherence between the 2 groups was consistent across all subgroups, and no significant interaction was observed. A trend of increased medication adherence in the intervention group was observed in the current drinker subgroup (8/33, 24%, vs 34/91, 37%; RR 0.72, 95% CI 0.65-0.80). However, the subgroup analysis did not indicate any significant interactions between medication adherence and stratification variables.

No study-related adverse events were recorded during this trial.

## Discussion

This is a prospective study, which directly compares web-based tertiary A-level hospital, WeChat-based secondary prevention with a traditional community, hospital-based cardiac rehabilitation program in patients with stable CAD.

### Principal Findings

Most patients with CAD had poor management of cardiovascular risk factors, and the percentage of participants who met the prescribed BP, BMI, LDL-C, and HbA_1c_ goals recommended were 71.31% (860/1206), 42.79% (516/1206), 27.2% (328/1206), and 74.88% (903/1206), respectively. Current smoking and alcohol consumption accounted for 25.54% (308/1206) and 15.92% (192/1206) of the total participants, respectively. A total of 31.01% (374/1206) of the participants had good medication adherence for the use of the 4 cardioprotective drugs. Cardioprotective medications included antiplatelet therapy, statins, β-blockers, and ACEIs/ARBs in 98.01% (1182/1206), 94.86% (1144/1206), 71.89% (867/1206), and 46.85% (565/1206) of patients, respectively. The current situation that uses secondary prevention is not ideal, especially regarding the participants’ blood lipids and blood glucose control. More than 73.60% (842/1144) of the participants in our study were taking statins to reduce their blood lipids, but their LDL-C levels remained high, suggesting that they needed more rigorous cholesterol treatment. A possible explanation is that the initial dose of the drug was too low, and the dose was not adjusted as soon as the treatment began, or failure to strengthen cholesterol management in a timely manner, such as the addition of ezetimibe and proprotein convertase subtilisin/kexin type 9 inhibitors. Our findings are in line with those of previous studies conducted in Europe, China, the United States, and other areas of the globe. Previous results from the European Action on Secondary and Primary Prevention by Intervention to Reduce Events V [21-24], Dyslipidemia International Study [25], Dyslipidemia International Study-China [26], the Prospective Urban Rural Epidemiology study [12], the prospective observational longitudinal registry of patients with stable CAD (CLARIFY [Prospective Observational Longitudinal Registry of Patients With Stable Coronary Artery Disease]) study [27], Report on Cardiovascular Health and Diseases Burden in China 2020 [28] confirmed that the current situation of secondary prevention in patients with coronary heart disease is concerning.

The most significant result of this study is that a WeChat-based intervention provided by a tertiary A-level hospital had no obvious advantage in improving patient adherence with the 4 cardioprotective medications compared with the traditional method. This finding is in contrast to a previous study [18], which suggested that the WeChat intervention can improve the medication adherence of patients. However, our study did not record the reasons for discontinuation of medication, and it is impossible to determine whether patients stopped medication because of a change in their disease or because of poor patient compliance. The WeChat remote intervention leads to better lifestyle improvements, including abstinence from smoking and alcohol consumption. The possible mechanism for the improved smoking cessation and alcohol use in participants in the intervention group includes several aspects, including how the relevant content is presented to the participants. All the messages sent out by our official WeChat account include smoking cessation and alcohol use content. Second, the participants were asked about smoking and alcohol consumption at each follow-up. Risk factors, including BP, LDL-C, blood glucose, and BMI, were more controlled by the traditional community follow-up, which may be explained by the fact that community hospital doctors were trained before the trial to improve their clinical skills.

### Limitations

This study had some limitations. First, this was an observational study. Bias may be introduced by variations in the baseline features of the 2 groups. Second, our study did not record the use of all antihypertensive drugs used in patients, so it was impossible to specifically analyze whether the reason for the better control of hypertension in participants in the control group was due to better compliance with antihypertensive drugs or an improved lifestyle. Third, because of the short follow-up period, it was difficult to assess the long-term consequences. In addition, some factors that might affect patient adherence, such as income and education, were not recorded in our study. Finally, participants were not asked to participate in designing messages and the intervention so that the interventions we offered may not fully meet their needs. Participants may be asked to contribute to the design of interventions in future trials.

### Comparison With Prior Work

According to a literature review by Farsi et al [29], multidimensional health care, which includes the integration of health care with social media and other kinds of communication, has been shown to be very effective. A systematic review by Indraratna et al [30] included 26 randomized controlled trials (n=6713). In patients with heart failure, mobile phone technologies were associated with lower hospitalization rates, and in patients with hypertension, mobile phone technologies significantly reduced the systolic BP [30]. A systematic review of 9 randomized controlled studies evaluated by Hamilton et al [31] confirmed that participants had high rates of participation, acceptance, use, and adherence to mobile health (mHealth). In addition, the health care provided by mHealth is just as effective as a traditional central health care and significantly improves the quality of life [31]. A few recent studies have demonstrated the feasibility and effectiveness of WeChat in chronic disease management, such as in hypertension and CAD. In a 30-day follow-up, Ni et al [17] discovered that the experimental group’s medication nonadherence score dropped more. Participants in that group were given an mHealth intervention created by combining 2 apps: *WeChat* and *BB Reminder*. The medication nonadherence score, heart rate, systolic BP, and diastolic BP were all included as outcome variables in this study. The remaining 3 outcomes were not examined owing to the short follow-up period (30 days) and small sample size of the study [17]. Dorje et al [18] created the Smartphone and Social Media–Based Cardiac Rehabilitation and Secondary Prevention in China program, which is a smartphone-based cardiac rehabilitation and secondary prevention program provided through the social media platform WeChat. The participants were monitored for a year in this randomized controlled study, which included 312 participants. The Smartphone and Social Media–Based Cardiac Rehabilitation and Secondary Prevention in China group improved substantially more in the 6-minute walk distance at 2 and 6 months than in the control group [18]. Participants in this group had better secondary outcomes, including knowledge of CAD total score, systolic BP, lipid profile, and cardioprotective drug compliance. However, they found no differences between groups in other secondary outcomes, such as current smoker, BMI, waist-to-hip ratio, psychosocial status, and quality of life. In addition, participants in the control group did not receive formal cardiac rehabilitation and secondary prevention, which may have led to an overestimation of the WeChat effect. BP control in the target range is an important strategy for secondary prevention of CAD. Several reports have shown that WeChat-based interventions are associated with a better control of hypertension. An investigation by Li et al [32] involving 464 patients with hypertension found that after 6 months of using WeChat for self-care, BP control was better in the intervention group. They built separate group chats according to the different risk factors and developed a punch in an innovative system to promote healthy behaviors. In a study by Xiao et al [33], participants reported feeling more willing to use and satisfied when using the WeChat platform for routine BP monitoring. Chang et al [34] examined participants’ experiences of physician-patient communication and peer interaction in a social media–based (WeChat) weight management program. The interactive nature of social media mitigates the practice of social support and social comparison and creates new forms of supervision [34]. However, such communication in a public group carries a potential privacy risk. In a study by Chen et al [35], 80 people were randomly allocated to 1 of 2 groups: intervention and control. The intervention group was given the entire Chinese smoking cessation plan, which was based on applicable guidelines. The features included projects that were used in a specific intervention program to help users plan and record good protocols to promote quitting smoking, promote smoking cessation games, provide information on smoking hazards, help users overcome impulse behaviors, evaluate the level of nicotine dependence and standardized lung health tests, and provide a social platform that encourages social support among users. A total of 25% (10/40) of the intervention participants and 5% (2/40) of the control participants (RR 5, 95% CI 1.2-21.4; *P*=.03) had biochemically validated cessation at 6 weeks. It has been suggested that using the WeChat platform for smoking cessation is a novel and acceptable intervention for smoking cessation. Zhang et al [36] found in their study that WeChat self-monitoring tended to increase the medication compliance of patients with ischemic stroke. However, owing to the study’s limited sample size, no significant conclusions could be drawn [36]. The study by Li et al [37] confirmed that the videoconference follow-up based on WeChat has better effectiveness, reliability, and higher user satisfaction and trust than the traditional telephone follow-up. The results of this study are consistent with those of our study, which verifies the feasibility of WeChat as a new method of long-term follow-up.

According to this study, WeChat-based telemedicine is particularly effective for lifestyle interventions. Owing to the COVID-19 crisis, it has been inconvenient and even impossible for patients with chronic diseases to receive outpatient follow-up. Remote follow-up can be used as an effective medical treatment.

We identified other problems in this study. First, participants in the intervention group often want consultation for their comorbidities, and our cardiac rehabilitation team may not be able to provide detailed explanations for their comorbidities, resulting in participants needing to go to the hospital. In the future, this problem can be solved by integrating a chronic disease management team to manage all comorbidities of participants. Second, some participants with persistent chest pain and who were suspected of having an MI still submitted a consultation through the WeChat platform rather than calling or going to the emergency center, which may lead to a delay in revascularization. Therefore, patient education may need to be enhanced when using these platforms, and it is important to inform the patient to be transported to the emergency department for additional care in case of life-threatening situations.

### Conclusions

Despite the prevalent use of cardioprotective medications, many patients with CAD fail to achieve ideal control of cardiovascular risk factors, as recommended by the guidelines. After initial treatment, the patient’s target should be monitored. The treatment regimen should be adjusted in time, and lifestyle interventions should be strengthened to try to control the risk factors and reach the target as soon as possible. Tertiary A-level hospital, WeChat-based intervention did not improve adherence to the 4 cardioprotective medications compared with the traditional method. Traditional community hospital follow-up was superior to WeChat remote follow-up in risk factor control, including BP, LDL-C, blood glucose, and BMI. The tertiary A-level hospital, WeChat-based intervention has a positive effect on improving lifestyle, such as quitting drinking and smoking, in patients with stable CAD and can be tried as a supplement to community hospital follow-up. Additional research on social media interventions aimed specifically at improving the lifestyle of patients with CAD is necessary.

## Appendix

Multimedia Appendix 1 The questionnaire that was sent remotely before the formal WeChat-based follow-up.

Multimedia Appendix 2 An example of a report that was sent remotely after the formal WeChat-based follow-up.

Multimedia Appendix 3 Clinical demographics of follow-up and lost to follow-up participants.

Multimedia Appendix 4 Standardized mean difference in the unadjusted, matched, and weighted cohorts.

Multimedia Appendix 5 Baseline characteristics of the propensity score-matched and weighted cohorts.

Multimedia Appendix 6 Primary and secondary outcomes at the 1-year follow-up (intervention vs control).

Multimedia Appendix 7 CONSORT-eHEALTH checklist (V 1.6.1).

## Abbreviations

ACEI/ARB: angiotensin-converting-enzyme inhibitor or angiotensin-receptor blocker
BP: blood pressure
CAD: coronary artery disease
CLARIFY: Prospective Observational Longitudinal Registry of Patients With Stable Coronary Artery Disease
DM: diabetes mellitus
GEE: generalized estimating equation
HbA_1c_: glycated hemoglobin A_1c_
IPTW: inverse probability of treatment weighting
LDL-C: low-density lipoprotein cholesterol
MI: myocardial infarction
mHealth: mobile health
PCI: percutaneous coronary intervention
PSM: propensity score matching
RR: relative risk

## References

[1] Zhou M, Wang H, Zeng X, Yin P, Zhu J, Chen W, Li X, Wang L, Wang L, Liu Y, Liu J, Zhang ME, Qi J, Yu S, Afshin A, Gakidou E, Glenn S, Krish VS, Miller-Petrie MK, Mountjoy-Venning WC, Mullany EC, Redford SF, Liu H, Naghavi M, Hay SI, Wang L, Murray CJ, Liang X (2019). Mortality, morbidity, and risk factors in China and its provinces, 1990-2017: a systematic analysis for the Global Burden of Disease Study 2017. Lancet.

[2] Task Force on Chinese Guidelines for the Prevention of Cardiovascular Diseases(2017), Editorial Board of Chinese Journal of Cardiology (2018). [Chinese guidelines for the prevention of cardiovascular diseases (2017)]. Zhonghua Xin Xue Guan Bing Za Zhi.

[3] Arnett DK, Blumenthal RS, Albert MA, Buroker AB, Goldberger ZD, Hahn EJ, Himmelfarb CD, Khera A, Lloyd-Jones D, McEvoy JW, Michos ED, Miedema MD, Muñoz D, Smith SC, Virani SS, Williams KA, Yeboah J, Ziaeian B (2019). 2019 ACC/AHA guideline on the primary prevention of cardiovascular disease: a report of the American College of Cardiology/American Heart Association task force on clinical practice guidelines. J Am Coll Cardiol.

[4] Piepoli MF, Hoes AW, Agewall S, Albus C, Brotons C, Catapano AL, Cooney M, Corrà U, Cosyns B, Deaton C, Graham I, Hall MS, Hobbs FD, Løchen ML, Löllgen H, Marques-Vidal P, Perk J, Prescott E, Redon J, Richter DJ, Sattar N, Smulders Y, Tiberi M, van der Worp HB, van Dis I, Verschuren WM, Binno S, ESC Scientific Document Group (2016). 2016 European Guidelines on cardiovascular disease prevention in clinical practice: The Sixth Joint Task Force of the European Society of Cardiology and Other Societies on Cardiovascular Disease Prevention in Clinical Practice (constituted by representatives of 10 societies and by invited experts) Developed with the special contribution of the European Association for Cardiovascular Prevention and Rehabilitation (EACPR). Eur Heart J.

[5] Yuhui Z, Peipei C, Quan W (2020). Study on accounting and analysis of curative expenditure on cardio-cerebrovascular diseases in China. Chin Circ J.

[6] Li J, Li X, Wang Q, Hu S, Wang Y, Masoudi FA, Spertus JA, Krumholz HM, Jiang L, China PEACE Collaborative Group (2015). ST-segment elevation myocardial infarction in China from 2001 to 2011 (the China PEACE-Retrospective Acute Myocardial Infarction Study): a retrospective analysis of hospital data. Lancet.

[7] Working Group on Coronary Artery Disease, National Center for Cardiovascular Quality Improvement (NCCQI) (2020). Clinical performance and quality measures for adults with acute ST-elevation myocardial infarction in China. China National Center for Cardiovascular Diseases.

[8] Shang P, Liu GG, Zheng X, Ho PM, Hu S, Li J, Jiang Z, Li X, Bai X, Gao Y, Xing C, Wang Y, Normand S, Krumholz HM (2019). Association between medication adherence and 1-year major cardiovascular adverse events after acute myocardial infarction in China. J Am Heart Assoc.

[9] Ni Z, Dardas L, Wu B, Shaw R (2019). Cardioprotective medication adherence among patients with coronary heart disease in China: a systematic review. Heart Asia.

[10] Kones R, Rumana U, Morales-Salinas A (2019). Confronting the most challenging risk factor: non-adherence. Lancet.

[11] Zhang M, Deng Q, Wang L, Huang Z, Zhou M, Li Y, Zhao Z, Zhang Y, Wang L (2018). Prevalence of dyslipidemia and achievement of low-density lipoprotein cholesterol targets in Chinese adults: a nationally representative survey of 163,641 adults. Int J Cardiol.

[12] Yusuf S, Joseph P, Rangarajan S, Islam S, Mente A, Hystad P, Brauer M, Kutty VR, Gupta R, Wielgosz A, AlHabib KF, Dans A, Lopez-Jaramillo P, Avezum A, Lanas F, Oguz A, Kruger IM, Diaz R, Yusoff K, Mony P, Chifamba J, Yeates K, Kelishadi R, Yusufali A, Khatib R, Rahman O, Zatonska K, Iqbal R, Wei L, Bo H, Rosengren A, Kaur M, Mohan V, Lear SA, Teo KK, Leong D, O'Donnell M, McKee M, Dagenais G (2020). Modifiable risk factors, cardiovascular disease, and mortality in 155 722 individuals from 21 high-income, middle-income, and low-income countries (PURE): a prospective cohort study. Lancet.

[13] Greenland P, Fuster V (2017). Cardiovascular risk factor control for all. J Am Med Assoc.

[14] Anderson L, Brown JP, Clark AM, Dalal H, Rossau HK, Bridges C, Taylor RS (2017). Patient education in the management of coronary heart disease. Cochrane Database Syst Rev.

[15] (2020). Science on WeChat. Nat Methods.

[16] Chen X, Zhou X, Li H, Li J, Jiang H (2020). The value of WeChat application in chronic diseases management in China. Comput Methods Programs Biomed.

[17] Ni Z, Liu C, Wu B, Yang Q, Douglas C, Shaw RJ (2018). An mHealth intervention to improve medication adherence among patients with coronary heart disease in China: development of an intervention. Int J Nurs Sci.

[18] Dorje T, Zhao G, Tso K, Wang J, Chen Y, Tsokey L, Tan B, Scheer A, Jacques A, Li Z, Wang R, Chow CK, Ge J, Maiorana A (2019). Smartphone and social media-based cardiac rehabilitation and secondary prevention in China (SMART-CR/SP): a parallel-group, single-blind, randomised controlled trial. Lancet Digit Health.

[19] Montalescot G, Sechtem U, Achenbach S, Andreotti F, Arden C, Budaj A, Bugiardini R, Crea F, Cuisset T, Di Mario C, Ferreira JR, Gersh BJ, Gitt AK, Hulot J, Marx N, Opie LH, Pfisterer M, Prescott E, Ruschitzka F, Sabaté M, Senior R, Taggart DP, van der Wall EE, Vrints CJ, Zamorano JL, Achenbach S, Baumgartner H, Bax JJ, Bueno H, Dean V, Deaton C, Erol C, Fagard R, Ferrari R, Hasdai D, Hoes AW, Kirchhof P, Knuuti J, Kolh P, Lancellotti P, Linhart A, Nihoyannopoulos P, Piepoli MF, Ponikowski P, Sirnes PA, Tamargo JL, Tendera M, Torbicki A, Wijns W, Windecker S, Knuuti J, Valgimigli M, Bueno H, Claeys MJ, Donner-Banzhoff N, Erol C, Frank H, Funck-Brentano C, Gaemperli O, Gonzalez-Juanatey JR, Hamilos M, Hasdai D, Husted S, James SK, Kervinen K, Kolh P, Kristensen SD, Lancellotti P, Maggioni AP, Piepoli MF, Pries AR, Romeo F, Rydén L, Simoons ML, Sirnes PA, Steg PG, Timmis A, Wijns W, Windecker S, Yildirir A, Zamorano JL, Task Force Members, ESC Committee for Practice Guidelines, Document Reviewers (2013). 2013 ESC guidelines on the management of stable coronary artery disease: the Task Force on the management of stable coronary artery disease of the European Society of Cardiology. Eur Heart J.

[20] Section of Interventional Cardiology of Chinese Society of Cardiology, Section of Atherosclerosis and Coronary Artery Disease of Chinese Society of Cardiology, Specialty Committee on Prevention and Treatment of Thrombosis of Chinese College of Cardiovascular Physicians (2018). [Guideline on the diagnosis and treatment of stable coronary artery disease]. Zhonghua Xin Xue Guan Bing Za Zhi.

[21] Kotseva K, De Backer G, De Bacquer D, Rydén L, Hoes A, Grobbee D, Maggioni A, Marques-Vidal P, Jennings C, Abreu A, Aguiar C, Badariene J, Bruthans J, Castro Conde A, Cifkova R, Crowley J, Davletov K, Deckers J, De Smedt D, De Sutter J, Dilic M, Dolzhenko M, Dzerve V, Erglis A, Fras Z, Gaita D, Gotcheva N, Heuschmann P, Hasan-Ali H, Jankowski P, Lalic N, Lehto S, Lovic D, Mancas S, Mellbin L, Milicic D, Mirrakhimov E, Oganov R, Pogosova N, Reiner Z, Stöerk S, Tokgözoğlu L, Tsioufis C, Vulic D, Wood D, EUROASPIRE Investigators* (2019). Lifestyle and impact on cardiovascular risk factor control in coronary patients across 27 countries: results from the European Society of Cardiology ESC-EORP EUROASPIRE V registry. Eur J Prev Cardiol.

[22] Jennings CS, Kotseva K, Bassett P, Adamska A, Wood D, ASPIRE-3-PREVENT Investigators (2020). ASPIRE-3-PREVENT: a cross-sectional survey of preventive care after a coronary event across the UK. Open Heart.

[23] Kotseva K, De Backer G, De Bacquer D, Rydén L, Hoes A, Grobbee D, Maggioni A, Marques-Vidal P, Jennings C, Abreu A, Aguiar C, Badariene J, Bruthans J, Cifkova R, Davletov K, Dilic M, Dolzhenko M, Gaita D, Gotcheva N, Hasan-Ali H, Jankowski P, Lionis C, Mancas S, Milićić D, Mirrakhimov E, Oganov R, Pogosova N, Reiner Z, Vulić D, Wood D (2020). Primary prevention efforts are poorly developed in people at high cardiovascular risk: a report from the European Society of Cardiology EURObservational Research Programme EUROASPIRE V survey in 16 European countries. Eur J Prev Cardiol.

[24] Blom DJ, Santos RD, Daclin V, Mercier F, Ruiz AJ, Danchin N, ICLPS study group (2020). The challenge of multiple cardiovascular risk factor control outside Western Europe: findings from the International ChoLesterol management Practice Study. Eur J Prev Cardiol.

[25] Gitt AK, Drexel H, Feely J, Ferrières J, Gonzalez-Juanatey JR, Thomsen KK, Leiter LA, Lundman P, da Silva PM, Pedersen T, Wood D, Jünger C, Dellea PS, Sazonov V, Chazelle F, Kastelein JJ, DYSIS Investigators (2012). Persistent lipid abnormalities in statin-treated patients and predictors of LDL-cholesterol goal achievement in clinical practice in Europe and Canada. Eur J Prev Cardiol.

[26] Wang F, Ye P, Hu D, Min Y, Zhao S, Wang Y, Mu Y, Yan X, Li Z, Wei Y, Li J, DYSIS-China Study Investigators (2014). Lipid-lowering therapy and lipid goal attainment in patients with metabolic syndrome in China: subgroup analysis of the Dyslipidemia International Study-China (DYSIS-China). Atherosclerosis.

[27] Ferrari R, Ford I, Greenlaw N, Tardif J, Tendera M, Abergel H, Fox K, Hu D, Shalnova S, Steg PG, CLARIFY Registry Investigators (2015). Geographical variations in the prevalence and management of cardiovascular risk factors in outpatients with CAD: data from the contemporary CLARIFY registry. Eur J Prev Cardiol.

[28] The Writing Committee of the Report on Cardiovascular Health and Diseases in China (2021). Report on cardiovascular health and diseases burden in China: an updated summary of 2020. Chin Circ J.

[29] Farsi D (2021). Social media and health care, Part I: literature review of social media use by health care providers. J Med Internet Res.

[30] Indraratna P, Tardo D, Yu J, Delbaere K, Brodie M, Lovell N, Ooi S (2020). Mobile phone technologies in the management of ischemic heart disease, heart failure, and hypertension: systematic review and meta-analysis. JMIR Mhealth Uhealth.

[31] Hamilton SJ, Mills B, Birch EM, Thompson SC (2018). Smartphones in the secondary prevention of cardiovascular disease: a systematic review. BMC Cardiovasc Disord.

[32] Li X, Li T, Chen J, Xie X, An X, Lv Y, Lin A (2019). A wechat-based self-management intervention for community middle-aged and elderly adults with hypertension in Guangzhou, China: a cluster-randomized controlled trial. Int J Environ Res Public Health.

[33] Xiao M, Lei X, Zhang F, Sun Z, Harris VC, Tang X, Yan L (2019). Home blood pressure monitoring by a mobile-based model in Chongqing, China: a feasibility study. Int J Environ Res Public Health.

[34] Chang L, Chattopadhyay K, Li J, Xu M, Li L (2021). Interplay of support, comparison, and surveillance in social media weight management interventions: qualitative study. JMIR Mhealth Uhealth.

[35] Chen J, Ho E, Jiang Y, Whittaker R, Yang T, Bullen C (2020). Mobile social network-based smoking cessation intervention for Chinese male smokers: pilot randomized controlled trial. JMIR Mhealth Uhealth.

[36] Zhang Y, Fan D, Ji H, Qiao S, Li X (2020). Treatment adherence and secondary prevention of ischemic stroke among discharged patients using mobile phone- and WeChat-based improvement services: cohort study. JMIR Mhealth Uhealth.

[37] Li L, Huang J, Wu J, Jiang C, Chen S, Xie G, Ren J, Tao J, Chan CC, Chen L, Wong AW (2020). A mobile health app for the collection of functional outcomes after inpatient stroke rehabilitation: pilot randomized controlled trial. JMIR Mhealth Uhealth.

