# Supplementary figures and images for: The Effect of a WeChat-Based Tertiary A-Level Hospital Intervention on Medication Adherence and Risk Factor Control in Patients With Stable Coronary Artery Disease: Multicenter Prospective Study
Source: JMIR Mhealth Uhealth. 2021 Oct 27;9(10):e32548. doi: 10.2196/32548 (PMC8581769; doi:10.2196/32548)

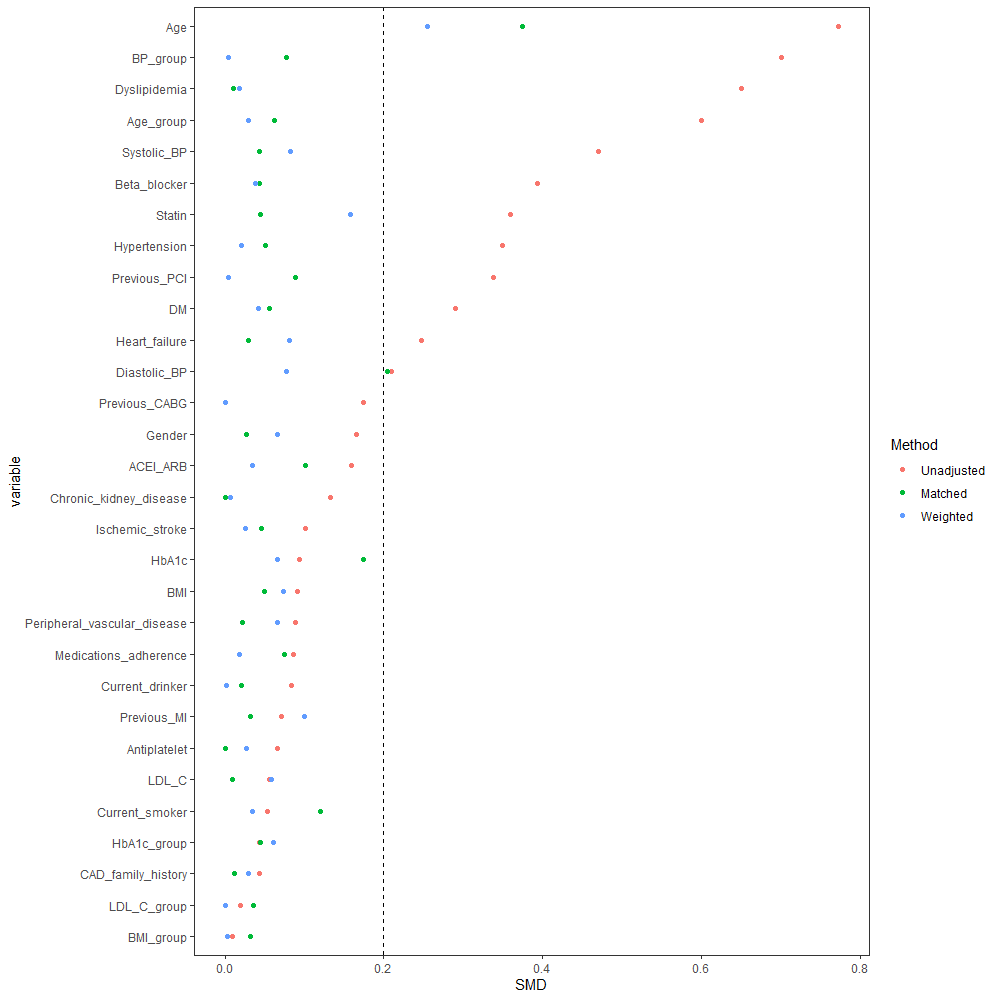

Supplement: Multimedia Appendix 4 [file mhealth_v9i10e32548_app4.png]
