# Supplementary material for: The Effect of a WeChat-Based Tertiary A-Level Hospital Intervention on Medication Adherence and Risk Factor Control in Patients With Stable Coronary Artery Disease: Multicenter Prospective Study
Source: JMIR Mhealth Uhealth. 2021 Oct 27;9(10):e32548. doi: 10.2196/32548 (PMC8581769; doi:10.2196/32548)
Supplement: Multimedia Appendix 6 [file mhealth_v9i10e32548_app6.docx]

Multimedia Appendix 6. Primary and secondary outcomes at the 1-year follow-up (intervention vs. control).

| Outcomes | | Baseline | | 1 year | | | | | | | | | | | | | | | |
| --- | --- | --- | --- | --- | --- | --- | --- | --- | --- | --- | --- | --- | --- | --- | --- | --- | --- | --- | --- |
|  | | Unadjusted | | Unadjusted | | | | Multivariable | | | | Matched | | | | Weighted | | | |
|  | | Propotion (intervention vs. control) | | Propotion (intervention vs. control) | | RR (95% CI) | *P value* | Propotion (intervention vs. control) | | RR (95% CI) | *P value* | Propotion (intervention vs. control) | | RR (95% CI) | *P value* | Propotion (intervention vs. control) | | RR (95% CI) | *P value* |
|  | | | | | | | | | | | | | | | | | | | |
| **Primary outcome** | | | | | | | | | | | | | | | | | | | |
|  | Medication adherence, n (%) | 211 (32.9) | 163 (28.9) | 172 (30.4) | 142 (27.4) | 0.99 (0.97-1.02) | .648 | 172 (30.4) | 142 (27.4) | 1.00 (0.90-1.12) | 1 | 62 (27.8) | 93 (35.6) | 1 (0.97-1.03) | 1 | 155.6 (28.0) | 133.4 (26.8) | 0.99 (0.97-1.01) | 0.383 |
| **Secondary outcomes** | | | | | | | | | | | | | | | | | | | |
|  | Antiplatelet, n (%) | 632 (98.4) | 550 (97.5) | 540 (95.6) | 494 (95.4) | 0.99 (0.98-1.00) | .137 | 540 (95.6) | 494 (95.4) | 1.00 (0.98-1.02) | 1 | 211 (94.6) | 259 (99.2) | 1.00 (0.99-1.01) | 1 | 531.3 (95.7) | 481.9 (96.8) | 1.00 (0.99-1.01) | 1 |
|  | β-blocker, n (%) | 514 (80.1) | 353 (62.6) | 441 (78.1) | 329 (63.5) | 0.98 (0.98-1.00) | .006 | 441 (78.1) | 329 (63.5) | 0.72 (0.96-1.04) | .145 | 159 (71.3) | 189 (72.4) | 1.00 (0.80-1.26) | 1 | 384.5 (69.3) | 346.0 (69.5) | 1.00 (0.56-1.77) | 1 |
|  | Statin, n (%) | 633 (98.6) | 511 (90.6) | 532 (94.2) | 479 (92.5) | 0.98 (0.97-0.99) | <.001 | 532 (94.2) | 479 (92.5) | 0.96 (0.94-0.97) | <.001 | 207 (92.8) | 254 (97.3) | 0.95 (0.94-0.97) | <.001 | 520.9 (93.8) | 476.8 (95.8) | 1.00 (1.00-1.00) | .996 |
|  | ACEI/ARB^a^, n (%) | 277 (43.1) | 288 (51.1) | 227 (40.2) | 258 (49.8) | 0.98 (0.96-0.99) | <.001 | 227 (40.2) | 258 (49.8) | 0.96 (0.93-0.99) | .01 | 88 (39.5) | 123 (47.1) | 0.96 (0.93-0.99) | 0.01 | 212.9 (38.4) | 218.8 (44.0) | 0.55 (0.49-0.61) | <.001 |
|  | Current smoker, n (%) | 171 (26.6) | 137 (24.3) | 44 (7.8) | 118 (22.8) | 0.48 (0.44-0.53) | <.001 | 44 (7.8) | 118 (22.8) | 0.48 (0.41-0.56) | <.001 | 19 (8.5) | 64 (24.5) | 0.48 (0.41-0.56) | <.001 | 38.3 (6.9) | 122.4 (24.6) | 0.44 (0.39-0.50) | <.001 |
|  | Current drinker, n (%) | 93 (14.5) | 99 (17.6) | 33 (5.8) | 91 (17.6) | 0.47 (0.42-0.54) | <.001 | 33 (5.8) | 91 (17.6) | 0.43 (0.35-0.50) | <.001 | 14 (6.3) | 43 (16.5) | 0.42 (0.36-0.50) | <.001 | 31.0 (5.6) | 80.6 (16.2) | 0.48 (0.40-0.57) | <.001 |
|  | Good control of hypertension, n (%) | 369 (57.5) | 491 (87.1) | 416 (73.6) | 486 (93.8) | 0.91 (0.90-0.94) | <.001 | 416 (73.6) | 486 (93.8) | 0.83 (0.76-0.91) | <.001 | 170 (76.2) | 206 (78.9) | 0.83 (0.76-0.91) | <.001 | 410.5 (73.9) | 459.5 (92.3) | 0.89 (0.84-0.93) | <.001 |
|  | 18.5<=BMI<25.0 kg/m^2^, n (%) | 276 (43.0) | 240 (42.6) | 237 (41.9) | 226 (43.6) | 0.95 (0.93-0.97) | <.001 | 237 (41.9) | 226 (43.6) | 0.95 (0.91-0.98) | .005 | 87 (39.0) | 104 (39.8) | 0.38 (0.31-0.45) | <.001 | 220.5 (39.7) | 203.0 (40.8) | 0.94 (0.91-0.98) | .001 |
|  | LDL-C^b^<1.8 mmol/L, n (%) | 172 (26.8) | 156 (27.7) | 198 (35.0) | 280 (54.1) | 0.79 (0.73-0.84) | <.001 | 198 (35.0) | 280 (54.1) | 0.81 (0.72-0.91) | <.001 | 65 (29.1) | 62 (23.8) | 0.81 (0.72-0.91) | <.001 | 174.1 (31.4) | 261.6 (52.5) | 0.77 (0.70-0.86) | <.001 |
|  | HbA_1c_^c^<7.0%, n (%) | 475 (74.0) | 428 (75.9) | 439 (77.7) | 484 (93.4) | 0.95 (0.94-0.97) | <.001 | 439 (77.7) | 484 (93.4) | 0.93 (0.90-0.95) | <.001 | 178 (79.8) | 197 (75.5) | 0.93 (0.90-0.95) | <.001 | 444.2 (80.0) | 465.9 (93.6) | 0.95 (0.93-0.97) | <.001 |

^a^ACEI/ARB: angiotensin-converting-enzyme inhibitor.
^b^LDL-C: low-density lipoprotein cholesterol.
^c^HbA_1c_: glycated hemoglobin.
